# Effectiveness of a periodontal re‐evaluation incentive program for patient compliance

**DOI:** 10.1002/jdd.13512

**Published:** 2024-04-08

**Authors:** Elisandra Reyes‐Perez, David J. Heinlein, Rachel C. Kearney, Fonda G. Robinson

**Affiliations:** ^1^ Division of Restorative and Prosthetic Dentistry The Ohio State University, College of Dentistry Columbus Ohio USA; ^2^ The Cincinnati Insurance Companies and The Cincinnati Life Insurance Company Fairfield Ohio USA; ^3^ Division of Dental Hygiene The Ohio State University, College of Dentistry Columbus Ohio USA

## 1 PROBLEM

Constant patient motivation is important in the success of periodontal treatment.^1^, ^2^ This issue was reflected in the dental school through student complaints of patients not returning for the periodontal re‐evaluation appointment. Clinical productivity data confirmed that students were assigned a sufficient number of patients with periodontal treatment needs to meet the educational expectations, but patients were not predictably returning for their periodontal re‐evaluation appointments following non‐surgical periodontal therapy. These critical re‐evaluation appointments provide students with the educational experiences to evaluate their treatment outcomes and plan for further maintenance or treatment. The inconsistent return rate for periodontal re‐evaluation appointments created challenges for students and a deficiency in this area of the clinical curriculum.

## 2 SOLUTION

**FIGURE 1:**
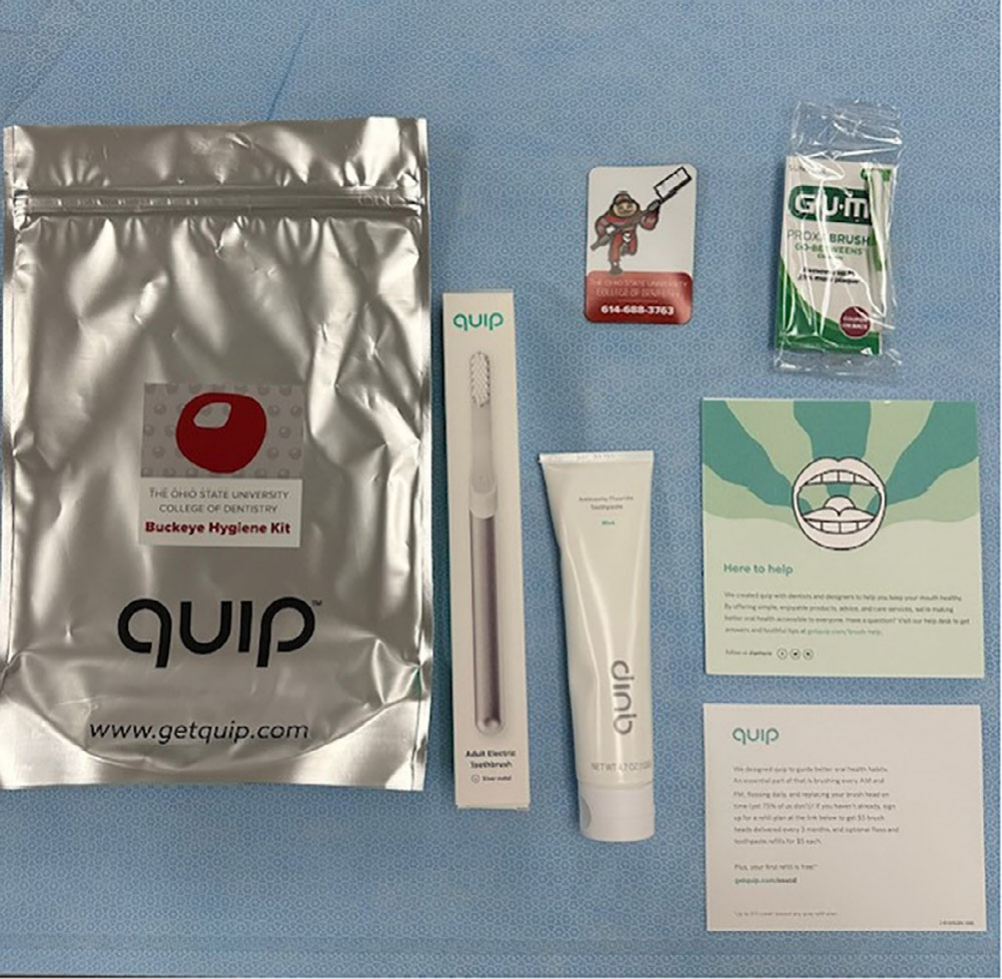
Buckeye Hygiene Kit.

In an effort to motivate patients to return for their “no charge” periodontal re‐evaluation appointment, an oral hygiene kit termed, “Buckeye Hygiene Kit,” was created. It included one Quip battery‐operated toothbrush, one Quip mint toothpaste, one individually packaged Proxabrush go‐betweens cleaners, and a university mascot magnet with the College of Dentistry's main telephone number (Figure 1).^3^, ^4^ The Buckeye Hygiene Kit was designed in compliance with patient inducement laws and exceptions.^5^, ^6^ A specific code was created in axiUm, the electronic health record, for treatment planning and completing the kit for tracking purposes.

**FIGURE 2:**
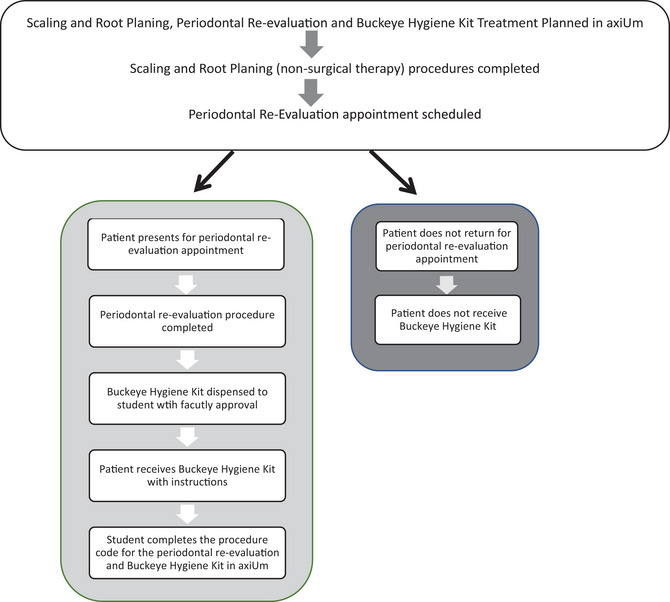
Buckeye Hygiene Kit distribution workflow.

The Buckeye Hygiene Kit code was included in the periodontal treatment plan. When a student completed the periodontal re‐evaluation appointment with a patient, the faculty member signed an approval form for the student to obtain the Buckeye Hygiene Kit from the dispensary. Dispensary staff members checked‐out the Buckeye Hygiene Kits to the students via the college's asset management tracking system A*STAR (Figure 2). There was a limit of one Buckeye Hygiene Kit per periodontal re‐evaluation patient.

To remind students to treatment plan and complete the Buckeye Hygiene Kit code in axiUm, a provider intervention warning pop‐up message was implemented. To enhance compliance, periodic reminders of the Buckeye Hygiene Kit process were included in the weekly student clinic electronic newsletter.

## 3 RESULTS

To ensure the process was followed appropriately by the students, the receiving and distribution manager generated weekly reports to compare the number of completed periodontal re‐evaluation codes in the electronic health record to the number of dispensed Buckeye Hygiene Kits through A*STAR. When inconsistencies existed, the receiving and distribution manager messaged the students and/or faculty members via axiUm either to reinforce the steps of the process or to request reconciliation of the patient's account.

A total of 488 kits were distributed in the student clinic 18 months following the implementation of the Buckeye Hygiene Kit. The program was deemed successful, increasing from a 66% rate of return for periodontal re‐evaluations in FY 2018 to a 79% rate of return for periodontal re‐evaluations in FY 2023 (Table 1). It is generally more cost‐effective motivating patients of record to return for the periodontal re‐evaluations versus marketing for new patients. This program supported timely and complete dental care while the procedures utilized were deemed to fall outside of proscribed inducement activities.

**TABLE 1:** Non‐surgical periodontal therapy patients rate of return FY 2018–FY 2023.

	FY 2018				FY 2019				FY 2020				FY 2021				FY 2022				FY 2023			
	SRP	Re‐Eval	Re‐Eval Rate	Kit	SRP	Re‐Eval	Re‐Eval Rate	Kit	SRP	Re‐Eval	Re‐Eval Rate	Kit	SRP	Re‐Eval	Re‐Eval Rate	Kit	SRP	Re‐Eval	Re‐Eval Rate	Kit	SRP	Re‐Eval	Re‐Eval Rate	Kit
July	54	33	61%		63	42	67%		41	30	73%		1				53	37	70%		30	26	87%	20
August	30	17	57%		54	37	69%		37	24	65%		5	4	80%		27	23	85%		21	18	86%	14
September	53	35	66%		59	44	75%		44	34	77%		26	14	54%		39	31	79%	1	39	31	79%	25
October	69	46	67%		78	63	81%		62	38	61%		36	21	58%		49	41	84%	2	52	42	81%	30
November	55	33	60%		52	40	77%		31	21	68%		29	19	66%		36	32	89%	2	53	38	72%	34
December	29	19	66%		27	21	78%		17	12	71%		36	29	81%		20	17	85%	2	33	28	85%	19
January	62	44	71%		57	45	79%		54	32	59%		34	27	79%		38	33	87%	13	46	39	85%	28
February	43	27	63%		58	42	72%		52	20	38%		39	32	82%		47	36	77%	22	64	52	81%	48
March	74	50	68%		56	37	66%		17	1	6%		67	47	70%		50	40	80%	31	61	45	74%	41
April	56	35	63%		66	34	52%						45	31	69%		55	31	56%	21	45	30	67%	28
May	47	33	70%		45	34	76%						38	29	76%		22	17	77%	10	41	32	78%	31
June	63	45	71%		65	49	75%		8				44	34	77%		32	27	84%	23	55	46	84%	40
Total	635	417	66%		680	488	72%		363	212	58%		400	287	72%		468	365	78%	127	540	427	79%	358

*Note*: SRP—count of unique patients who had SRP (if patient has multiple SRPs the patient is only counted once in the month the most recent one occurred). RE‐Eval—count of unique patients who eventually came in for a Re‐Eval following the SRP over the next year. Re‐Eval Rate—count of Re‐Eval/count of SRP = Re‐Eval Rate. Kit—count of those who had a Re‐Eval that also received a Buckeye Hygiene Kit. Gray shadow—COVID‐19 pandemic shutdown. No data available.
